# Comparing hemodynamic and cardiorespiratory responses during six-minute walk and step tests in mild acute COVID-19

**DOI:** 10.1038/s41598-026-41337-1

**Published:** 2026-02-23

**Authors:** Aldair Darlan Santos-de-Araújo, Daniela Bassi-Dibai, Renan Shida Marinho, Shane A. Phillips, Ross Arena, Audrey Borghi-Silva

**Affiliations:** 1 Cardiopulmonary Physical Therapy Laboratory, Physical Therapy Department, Federal University of São Carlos, Rodovia Washington Luiz, São Carlos, SP 13565-905 Brazil; 2 Paranaense University (UNIPAR), Francisco Beltrão, PR Brazil; 3 Department of Dentistry, CEUMA University, São Luís, MA Brazil; 4 Postgraduate Program in Management in Health Programs and Services, CEUMA University, São Luís, MA Brazil; 5 Postgraduate Program Inter-units of Bioengineering, University of São Paulo, São Carlos, SP Brazil; 6 Department of Physical Therapy, College of Applied Health Sciences, University of Illinois at Chicago, Chicago, IL USA; 7 Healthy Living for Pandemic Event Protection (HL-PIVOT) Network, Chicago, IL USA; 8 HealthPartners Institute, Minneapolis, MN USA

**Keywords:** Functional capacity, Six-minute step test, Six-minute walking test, COVID-19, Cardiovascular diseases, Respiratory tract diseases, Respiration, Physiology, Cardiology, Diseases, Signs and symptoms, Respiratory signs and symptoms

## Abstract

The six-minute walking test (6MWT) and six-minute step test (6MST) are valuable tools for assessing functional capacity and predicting outcomes in individuals suffering from mild COVID-19. This study aims to evaluate functional capacity and oxygen uptake (${\dot{\text {V}}}{{\mathrm{O}}_{\mathrm{2}}}$) during both the 6MWT and 6MST, to examine hemodynamic and cardiorespiratory responses and identify predictive factors influencing performance and ${\dot{\text {V}}}{{\mathrm{O}}_{\mathrm{2}}}$ (mL kg⁻¹ min⁻¹). This is a cross-sectional study including adults with mild COVID-19 symptoms within 6 weeks of a positive RT-PCR test. Participants were assessed for anthropometrics, handgrip strength, physical activity levels, pulmonary function, and performance on the 6MWT/6MST. Cardiorespiratory data were collected using a portable gas analyzer. Statistical analyses were conducted to compare the two tests, and regression models were used to identify predictive factors for performance and ${\dot{\text {V}}}{{\mathrm{O}}_{\mathrm{2}}}$ peak (mL kg⁻¹ min⁻¹). Forty volunteers (57% female) participated, with a mean age of 35 ± 12 years and BMI of 27.55 ± 5.66 kg/m^2^. Mean 6MWT distance was 473 ± 97 m (82 ± 18% predicted) and mean 6MST was 144 ± 27 steps (81 ± 16% predicted). Significant differences were found in hemodynamic responses with the 6MST eliciting higher heart rate (HR; *p* < 0.001), systolic blood pressure (SBP; *p* < 0.001), and ratings of dyspnea and lower limb fatigue on the Borg scale (*p* < 0.001 and *p* = 0.015, respectively). (Regression analyses revealed factors that predicted performance and ${\dot{\text {V}}}{{\mathrm{O}}_{\mathrm{2}}}$ peak (mL kg⁻¹ min⁻¹) for both tests, with models explaining 46–59% of variance for the 6MST and 12–40% for the 6MWT. The 6MST and 6MWT elicit distinct physiological responses, with the 6MST imposing greater hemodynamic and cardiorespiratory responses. Pulmonary function and body composition significantly enhance predictive models for functional performance and ${\dot{\text {V}}}{{\mathrm{O}}_{\mathrm{2}}}$ peak (mL kg⁻¹ min⁻¹) in both tests.

**Supplementary Information:**

The online version contains supplementary material available at 10.1038/s41598-026-41337-1.

## Introduction

Understanding the coupling of hemodynamic and cardiorespiratory responses, along with functional and exercise outcomes, provides insights into both the health and integrated functioning of the cardiovascular, respiratory, and musculoskeletal systems^1–3^. Achieving homeostasis relies on a dynamic balance of regulatory mechanisms, which can be disrupted by external and internal factors^4^. In this context, SARS-CoV-2, through its affinity for specific receptors in these tissues, can impair physiological functioning, triggering a pathophysiological cascade that may result in persistent or even irreversible dysfunction, often independent of COVID-19 severity^5,6^.

The inability to adequately regulate COVID-19–induced disturbances in tissue perfusion and gas exchange may lead to reduced exercise tolerance, lower oxygen uptake (${\dot{\text {V}}}{{\mathrm{O}}_{\mathrm{2}}}$), increased perceived exertion, altered autonomic modulation, and delayed exercise recovery^7–9^. This decline in cardiorespiratory fitness appears to result from the combined influence of age, disease severity, and time since infection, and residual respiratory symptoms. Notably, such alterations are not restricted to severe cases, as even mild infections can produce persistent disturbances in cardiopulmonary and autonomic responses to exercise^10,11^.

Although the cardiopulmonary exercise testing (CPET) is considered the gold standard for assessing exercise capacity and identifying the physiological mechanisms underlying exercise intolerance, its clinical application is often limited by complex logistics^12^. Consequently, field tests such as the six-minute walk test (6MWT) and six-minute step test (6MST) have been increasingly employed to assess these post-COVID sequelae, given their simplicity, low cost, and reliability^13,14^. Both tests have demonstrated strong clinical applicability across diverse populations, including healthy individuals and those with chronic obstructive pulmonary disease, heart failure, diabetes mellitus, and post-COVID conditions^2,15–20^. Beyond detecting functional impairments and monitoring disease progression, parameters derived from these submaximal tests have been increasingly employed in rehabilitation programs to individualize exercise prescription and evaluate therapeutic efficacy. The choice between tests depends on clinical conditions, assessment goals, and available resources^16,17,19,21–27^.

While the 6MWT assesses functional capacity related to daily activities, the 6MST introduces an intermittent resistive component recruiting more lower limb muscles to overcome gravity with each step^2,18–20,28,29^. The different types of muscle contractions, predominantly eccentric-concentric in the 6MST and isotonic in the 6MWT, affect cardiac output, blood flow redistribution, and ventilatory demands^30^. In the 6MWT, the ventilatory response increases progressively with aerobic demand, while in the 6MST, it is additionally influenced by the abrupt increase in metabolic load and peripheral muscle fatigue^1,31,32^.

The short-term impact of mild COVID-19 (≤ 6 weeks post-symptom onset) on hemodynamic and cardiorespiratory responses during the 6MWT and 6MST, as well as the physiological and clinical interpretability of these submaximal tests, remains underexplored. Given that even mild infection can lead to persistent impairments in exercise tolerance^10^, investigating these differences could provide valuable insights into test selection, safety, and functional assessment in this population, ultimately informing future clinical decision-making and rehabilitation strategies. Therefore, this study primarily aimed to evaluate functional capacity and ${\dot{\text {V}}}{{\mathrm{O}}_{\mathrm{2}}}$ during both tests in individuals with mild COVID-19, while also examining hemodynamic and cardiorespiratory responses and identifying clinical predictors of ${\dot{\text {V}}}{{\mathrm{O}}_{\mathrm{2}}}$ and test performance.

## Methodology

### Study design

This is a cross-sectional observational study. The guideline of the Strengthening Reporting of Observational Studies in Epidemiology was followed to ensure the appropriate reporting of the study^33^. It was conducted at the Federal University of São Carlos (São Carlos, SP, Brazil) and the University Hospital of the Federal University of São Carlos (HU-UFSCar/EBSERH). The study was approved by the institutional Research Ethics Committee (report number: 5.499.064) and conducted in accordance with the Declaration of Helsinki. All participants provided written informed consent prior to participation.

### Sample size

A post-hoc power analysis was performed for VO₂ peak to evaluate the adequacy of the sample size. The observed VO₂ values observed were 20.37 ± 6.75 for the 6MST and 16.71 ± 5.92 for the 6MWT, with a correlation of *r* = 0.5439 between the tests. Using a paired t-test (two-tailed), the pooled standard deviation of the difference was 6.06, resulting in a Cohen’s d of 0.604, which represents a moderate effect size. With an α of 0.05, the study achieved a post-hoc power of 95.1% to detect the observed difference in VO₂. Other cardiorespiratory outcomes were analyzed as exploratory measures.

### Participants

Participants of both sexes, aged 18 years or older, with infection diagnosed by RT-PCR for SARS-CoV-2, occurring up to 6 weeks before the start of the investigation, were recruited between April 2022 and May 2023 using a convenience sampling approach. Recruitment was conducted through multiple strategies to ensure broad community reach. They were invited to take part via digital media announcements (e.g., university website and social networks), printed flyers disseminated on campus and in clinical environments, and direct contact in outpatient clinics associated with the university hospital. The National Institutes of Health (NIH) COVID-19 treatment guideline was adopted^34^ to characterize mild COVID-19 symptoms: presence of signs and symptoms of the disease, such as fever, cough, sore throat, malaise, pain headache, muscle pain, nausea, vomiting, diarrhea, loss of taste and smell, peripheral oxygen saturation (≥ 95%).

Participants were not included in the study if they had been diagnosed with moderate to severe COVID-19 symptoms, hospitalized as a result of COVID-19 infection, experienced a myocardial infarction, received a pacemaker or metal implant, had a history of heart disease, unstable angina, uncontrolled hypertension or diabetes, chronic obstructive pulmonary disease or other respiratory diseases, neoplasms, cognitive impairment, reported illicit drug use, or were pregnant.

### Risk factors, medications and vaccination status

The collection of information on risk factors, medications of vaccination status of the participants was carried out from two main sources: medical records and the patients’ own reports. If possible, the information was taken from the medical records, which provide precise and detailed data from the healthcare team. When these records were unavailable or did not provide sufficient information, the participants’ self-report was used to supplement the necessary data.

### Anthropometrics variables

The participants’ height (m) was determined using a stadiometer (Welmy R-110, Santa Bárbara do Oeste, São Paulo, Brazil). Body composition was assessed with a bioelectrical impedance analyzer (InBody 720, Seoul, South Korea)^35^, measuring body mass in kilos (kg), body fat mass (kg), skeletal muscle mass (kg), basal metabolic rate in kilocalories (Kcal), right lower limb fat in percentage (%), left lower limb fat (%), body fat (%), and body mass index [BMI(kg/m^2^)].

Prior to this assessment, some recommendations were provided to ensure accurate measurements. Participants were advised to: (1) fast for at least four hours before arriving at the laboratory; (2) wear light clothing; (3) remove any metal accessories in contact with the body; (4) empty their bladder before the test; (5) refrain from consuming alcohol for 12 h before the evaluation; and (6) avoid intense physical activity on the day before the assessment.

### Handgrip strength

Handgrip strength was assessed using a hydraulic hand dynamometer (SAEHAN Corporation, Changwon, South Korea). The participant remained seated with the elbow flexed at 90° and the forearm positioned alongside the body, while the hand remained in a neutral position, holding the dynamometer. Next, the participant was instructed to squeeze the device with maximum strength while receiving verbal encouragement during the execution. At least three measurements were taken on the dominant upper limb, with a one-minute rest interval between them, provided that the variation between attempts was less than 10%. The average of the three measurements was considered for analysis^36^.

### Level of physical activity: Baecke questionnaire

This is a self-administered questionnaire based on self-report, designed to assess physical activity performed in the past 12 months. It includes 16 items, categorized into three domains: occupational (items 1 to 8), sports (items 9 to 12), and leisure (items 13 to 16). Responses are rated using a Likert scale ranging from 1 to 5^37,38^. The final score for each domain ranges from 1 to 5, with higher scores indicating higher levels of physical activity^37,38^.

### Modified medical research council (mMRC)

This 5-item questionnaire allows patients to rate their level of disability, showing how dyspnea impacts their mobility^39^. They indicate the intensity of dyspnea subjectively, choosing a value between 0 and 4. Higher scores on the MMRC indicate more impairment in daily activities due to dyspnea.

### Pulmonary function

The pulmonary function test was performed using a whole-body plethysmograph (Masterscreen Body, Mijnhardt/Jäger, Würzburg, Germany), and the following assessments were conducted: spirometry, plethysmography, lung diffusion capacity, and respiratory muscle strength. The examination was carried out by a trained researcher, and following the recommendations of the American Thoracic Society and European Respiratory Society (ATS/ERS)^40,41^. Measurements were performed with participants seated, wearing a nose clip, and using a disposable mouthpiece to prevent air leaks. Recorded parameters include: forced vital capacity (FVC) in liters (L) and (%), forced expiratory volume in the first second (FEV_1_) in (L) and (%), FEV_1_/FVC ratio, total lung capacity (TLC) in (%), diffusing capacity of the lungs for carbon monoxide (DLco) using the single-breath (SB) method in [ml/(min mmHg)] and (%), carbon monoxide transfer coefficient (Kco) in [ml/(min mmHg)]

Respiratory muscle strength was assessed using maximal inspiratory pressure (MIP, %) and maximal expiratory pressure (MEP, %)^42^ using the same equipment for pulmonary function. Following ATS/ERS recommendations, at least three technically acceptable and reproducible maneuvers (≤ 10% variation between the two highest values) were obtained for each variable, and the highest value was retained for analysis. Measured pressures were compared with predicted reference values for the Brazilian population^43^.

### Six-minute walk test (6MWT) and six-minute step test (6MST) protocols

Both tests followed a protocol that consisted of 4 min of rest, with two minutes seated and two minutes standing, respectively, six minutes of test performance, and six minutes of seated recovery. The tests were conducted on the same day, with at least thirty minutes of rest between them to minimize potential fatigue effects. All participants performed the 6MWT first, followed by the 6MST. All participants were informed about the nature of performing both tests before the start of the protocols. Vital signs were collected in the resting seated position before the start of the tests and at the peak of the tests: heart rate in beats per minute (bpm), SpO_2_ in %, systolic blood pressure (SBP) and diastolic blood pressure (DBP) in millimeters of mercury (mmHg). Additionally, perceived exertion for dyspnea, and leg fatigue was assessed by rating of perceived exertion (RPE) using the Borg 10 scale.

Regarding the 6MWT, the participants were instructed to walk as much as possible for six minutes, while for the 6MST, the participants were instructed to go up and down a single step with a height of 20 cm in a self-paced manner as many times as possible within the allotted time. Participants were informed that they could slow down, stop, and rest as needed, but they should resume the test as soon as possible. Verbal encouragement was given each minute with an account of remaining time to complete both tests. The step numbers and walking distance were recorded. Vital signs (HR, SBP, DBP, and SpO_2_) and RPE (dyspnea and leg fatigue) were assessed before the tests, likewise collected at the end of the tests.

Although the test is generally considered submaximal, certain criteria were established to stop the test in order to ensure the participants’ safety: SpO2 ≤ 87%, RPE > 7 due to dyspnea or lower limb fatigue on the Borg category ratio 10 scale, or the presence of dizziness, vertigo, or nausea symptoms^2^. HRmax was calculated using the formula: $\:HRmax=208-\left(0.7\:x\:age\right)$^44^. Recovery HR was assessed at the first, third, and sixth minutes after the test, considering both the absolute values and the difference relative to the peak HR.

For the 6MWT, functional capacity was estimated using the formula by Britto et al., which explains 62% of the test variation in Brazilian population^45^: 6MWT = 356.658 – (2.303 × age) + (36.648 × gender [0, female; 1, male]) + (1.704 × height [cm]) + (1.365 ∆HR). Age is in years, sex is 0 for female and 1 for male, and height is in centimeters (cm). For the 6MST, we used the formula developed for the Brazilian population^18^: 6MST = 106 + (17.02 × [0: woman; 1: man]) + (− 1.24 × age) + (0.8 × height [cm]) + (− 0.39 × weight [kg]), which explains 42% of the performance.

### Oxygen uptake and ventilatory parameters

The cardiorespiratory and metabolic responses were assessed using a portable telemetric gas analysis system (Oxycon Mobile Mijnhardt/Jager, Würzburg, German). Breath-by-breath analysis ventilatory expired gas analysis was obtained throughout the tests. The following data were recorded during all 6MST and 6MWT protocols: ${\dot{\text {V}}}{{\mathrm{O}}_{\mathrm{2}}}$ in milliliters per minute (mL min) and corrected by weight (mL kg⁻¹ min⁻¹), ${\dot{\text {V}C}}{{\mathrm{O}}_{\mathrm{2}}}$ (mL min), minute ventilation in liters per minute (${{\dot{\text {V}}}_{\mathrm{E}}}$, L/min), respiratory rate (RR) in breaths per minute (breaths min⁻¹), and the respiratory exchange ratio (RER)^1^. Gas exchange variables were calculated as the mean values obtained during the final 20 s of each minute throughout the test.

### Statistical analysis

Data are presented as mean ± standard deviation (SD), median [interquartile range (IQR 25–75)], or absolute value and %. The normality of the data was assessed using the Shapiro-Wilk test^46^. Depending on the data distribution, the paired t-test or Wilcoxon test were used to compare 6MST and 6MWT. We use simple linear regression models to investigate the relationship between an independent variable (predictor) and a dependent variable (outcome). Variables with a *p* < 0.20 in univariate analysis were allocated to multiple linear regression using the forward selection method^47^. The final multiple regression model was then adjusted by enter method to improve the explanation of variance (adjusted R^2^).

The homoscedasticity of the residuals was evaluated using a scatter plot of the unstandardized residuals against the fitted values. This analysis aimed to confirm that the variance of the residuals remains constant across the fitted values, thereby ensuring compliance with the homoscedasticity assumption required for regression analysis^48^. The Durbin-Watson test was applied to detect autocorrelation in the residuals of the regression model, with values below 2 suggesting positive autocorrelation and values above 2 indicating negative autocorrelation^49^.

Collinearity among independent variables was examined through the Variance Inflation Factor (VIF) and tolerance values, considering VIF values under 10 and tolerance values near 1 as acceptable thresholds to rule out collinearity^50^. We considered a number of 10 participants for each independent variable added to the final multiple regression model^48,51^. A *p* < 0.05 was adopted to demonstrate statistical difference. The effect size was calculated based on the Cohen’s d (parametric distribution) or Pearson’s r (r) (non-parametric distribution), according to the website: https://www.psychometrica.de/effect_size.html. The following interpretation was considered for Cohen’s d: 0.2 (small), 0.5 (moderate), and > 0.8 (large) effect size^52^. For Pearson’s r, values close to 0.10 are considered small, those around 0.30 are moderate, and values greater than 0.50 are large effect sizes^52^. All analysis were performed using GraphPad Software, Inc. (2019). *GraphPad Prism* (version 8.0.1). San Diego, CA https://www.graphpad.com.

## Results

Initially, 46 participants were recruited; however, six did not complete all the assessments, leaving a final sample of 40 participants for analysis.

### Demographics and clinical characteristics

The sample was predominantly female (57%), with a mean age of 35 ± 12 years and a BMI of 27.55 ± 5.66 kg/m². Pulmonary function, assessed by spirometry, showed a mean FEV_1_ of 3.26 ± 0.70 L (94.58 ± 12.76% predicted) and an FEV_1_/FVC ratio of 0.82 ± 0.07. Pulmonary diffusion capacity, measured using the single-breath method (DLcoSB), was 25.39 ± 7.69 ml/(min mmHg), (84.43 ± 15.71% predicted). Respiratory muscle strength was 93.05 ± 29.37% of the predicted value for maximal inspiratory pressure (MIP) and 79.38 ± 21.11% for maximal expiratory pressure (MEP). Handgrip strength (kgf) in the dominant limb averaged 28.32 ± 7.34 kgf. Among risk factors, 22% had systemic arterial hypertension, 7% had dyslipidemia, and 7% had diabetes mellitus. The most reported symptoms were cough (75%), sore throat (67%), fever (57%), and headache (57%). Additionally, 32% of individuals reported breathlessness, and 52% reported fatigue. Regarding dyspnea, 35% of participants had an mMRC score of 1, while 30% had a score of 3. Detailed sample characteristics are shown in Table 1.

**Table 1:** Clinical and anthropometric characteristics of participants included in the analyses.

Variables	All included (40)
Age (years)	35.00 ± 12.00
*Gender*	
Male	17.00 (42.50)
Female	23.00 (57.50)
*Height (m)*	1.69 ± 0.10
*Bioelectrical impedance analysis*	
Body mass (kg)	79.11 ± 17.38
Body fat mass (kg)	25.21 ± 12.55
Skeletal muscle mass (kg)	30.33 ± 6.92
Basal metabolic rate (Kcal)	1534.97 ± 247.47
Body fat (%)	31.28 ± 11.23
Right lower limb fat (%)	157.86 ± 81.56
Left lower limb fat (%)	160.52 ± 78.48
BMI (kg/m²)	27.55 ± 5.66
Pulmonary function	
*Spirometry*	
FEV_1_ (L)	3.26 ± 0.70
FEV_1_ (%, predicted)	94.58 ± 12.76
FVC (L)	3.98 ± 0.87
FVC (%, predicted)	97.53 ± 12.13
FEV_1_/FVC	0.82 ± 0.07
*Diffusion*	
DLcoSB [ml/(min mmHg)]	25.39 ± 7.69
DLcoSB (%, predicted)	84.43 ± 15.71
Kco [ml/(min mmHg L)]	5.04 ± 0.78
Kco (%, predicted)	99.58 ± 15.48
*Body plethysmography*	
TLC (%, predicted)	92.93 ± 16.71
*Respiratory muscle strength*	
MIP (%, predicted)	93.05 ± 29.37
MEP (%, predicted)	79.38 ± 21.11
Handgrip strength (Kgf) (dominant member)	28.32 ± 7.34
*Level of physical activity (Baecke questionnaire)*	
Occupational domain	2.89 ± 0.51
Sports domain	2.55 ± 0.77
Leisure domain	2.38 ± 0.80
Final score	7.82 ± 1.45
*Risk factors*	
Systemic arterial hypertension	9.00 (22.50)
Osteoporosis	1.00 (2.50)
Stress	8.00 (20.00)
Thyroid dysfunction	2.00 (5.00)
Dyslipidemia	3.00 (7.50)
Diabetes mellitus	3.00 (7.50)
Depression	7.00 (17.50)
Former smoking	2.00 (5.00)
*mMRC*	
0	2.00 (5.00)
1	14 (35.00)
2	10.00 (25.00)
3	12.00 (30.00)
4	2.00 (5.00)
*Symptomatology*	
Fever	23.00 (57.50)
Cough	30.00 (75.00)
Sore throat	27.00 (67.50)
Breathlessness	13.00 (32.50)
Diarrhea	10.00 (25.00)
Nausea	6.00 (15.00)
Vomiting	5.00 (12.50)
Headache	23.00 (57.50)
Runny nose	22.00 (55.00)
Asthenia	21.00 (52.50)
Chills	13.00 (32.50)
Nasal congestion	25.00 (62.50)
Anosmia	6.00 (15.00)
Ageusia	5 (12.50)
*Vaccination status*	
No	1.00 (2.50)
Yes	
Two doses	8.00 (20.00)
Three doses	31.00 (77.50)

Kg: kilos; m: meter; BMI: body mass index; Kcal: kilocalories; %: percentage; mMRC: modified medical research council; FVC: forced vital capacity; L: liter; %: percentage; FEV_1_: forced expiratory volume in first second; FEV_1_/FVC: ratio between forced vital capacity and forced expiratory volume in first second; DLcoSB: Diffusing capacity of the lung for carbon monoxide single-breath; mmHg: millimeters of mercury; Kco: transfer coefficient for carbon monoxide; TLC: thoracic lung capacity; MPI: maximum inspiratory pressure; MEP: maximum expiratory pressure; kgf: kilos-force.

### Functional performance

Functional performance results are listed in Table 2. For the 6MWT, the mean distance walked was 473 ± 97 m (82 ± 18% of the predicted distance according Albuquerque et al.^45^). For the 6MST, mean of 144 ± 27 steps was achieved, which corresponded to 81 ± 16%.of the predicted value based on the criteria of Britto et al^18^. No participant interrupted the test or exhibited limiting signs or symptoms that required premature termination.

**Table 2:** Functional capacity and hemodynamics responses through 6MWT and 6MST.

Variables	6MWT	6MST	*P* value	Effect size
Steps from 6MST	–	144 ± 27	–	–
% Predict by Albuquerque et al. (2022)	–	81 ± 16	–	–
Distance walked (m)	473 ± 97	–	–	–
% Predict by Britto et al. (2013)	82 ± 18	–	–	–
HR (bpm) rest	81 ± 13	84 ± 14	0.636	0.222
HR (bpm) peak	128 ± 22	158 ± 21	< 0.001*	1.395
HR (bpm) rec 1′	− 25 ± 14	− 35 ± 15	0.001*	0.689
HR (bpm) rec 3′	− 34 ± 15	− 53 ± 13	< 0.001*	1.354
HR (bpm) rec 6’	− 39 ± 15	− 60 ± 14	< 0.001*	1.447
%HRmax	69 ± 11	85 ± 8	< 0.001*	1.664
SBP (mmHg) rest	113 (102–129)	112 (110–129)	0.182	0.172
SBP (mmHg) peak	132 (122–145)	149 (134–168)	< 0.001*	0.329
DBP (mmHg) rest	77 ± 9	76 ± 9	0.351	0.111
DBP (mmHg) peak	79 ± 9	81 ± 12	0.266	0.189
SpO_2_ (%) rest	97 (96–98)	96 (96–98)	0.404	0.003
SpO_2_ (%) peak	96 (95–97)	96 (95–98)	0.171	0.034
BORG Dyspnea peak	1 (0.50–3)	3 (1–3)	< 0.001*	0.180
BORG fatigue lower limbs peak	1 (0.50–3)	3 (0.50–5)	0.015*	0.194

Values are mean ± Standard Deviation or median and interquartile range. 6MWT: six-minute walking test; 6MST: six-minute step test; %: percentage; m: meter; HR: heart rate; bpm: beats per minute; rec: recovery; ‘: minute; max: maximum; SBP: systolic blood pressure; mmHg: millimeters of mercury; DBP: diastolic blood pressure; SpO_2_: peripheral oxygen saturation. *Statistical difference (p < 0.05) between 6MST and 6MWT for the paired t-test or Wilcoxon test.

### Cardiorespiratory and hemodynamics responses

**Fig. 1:**
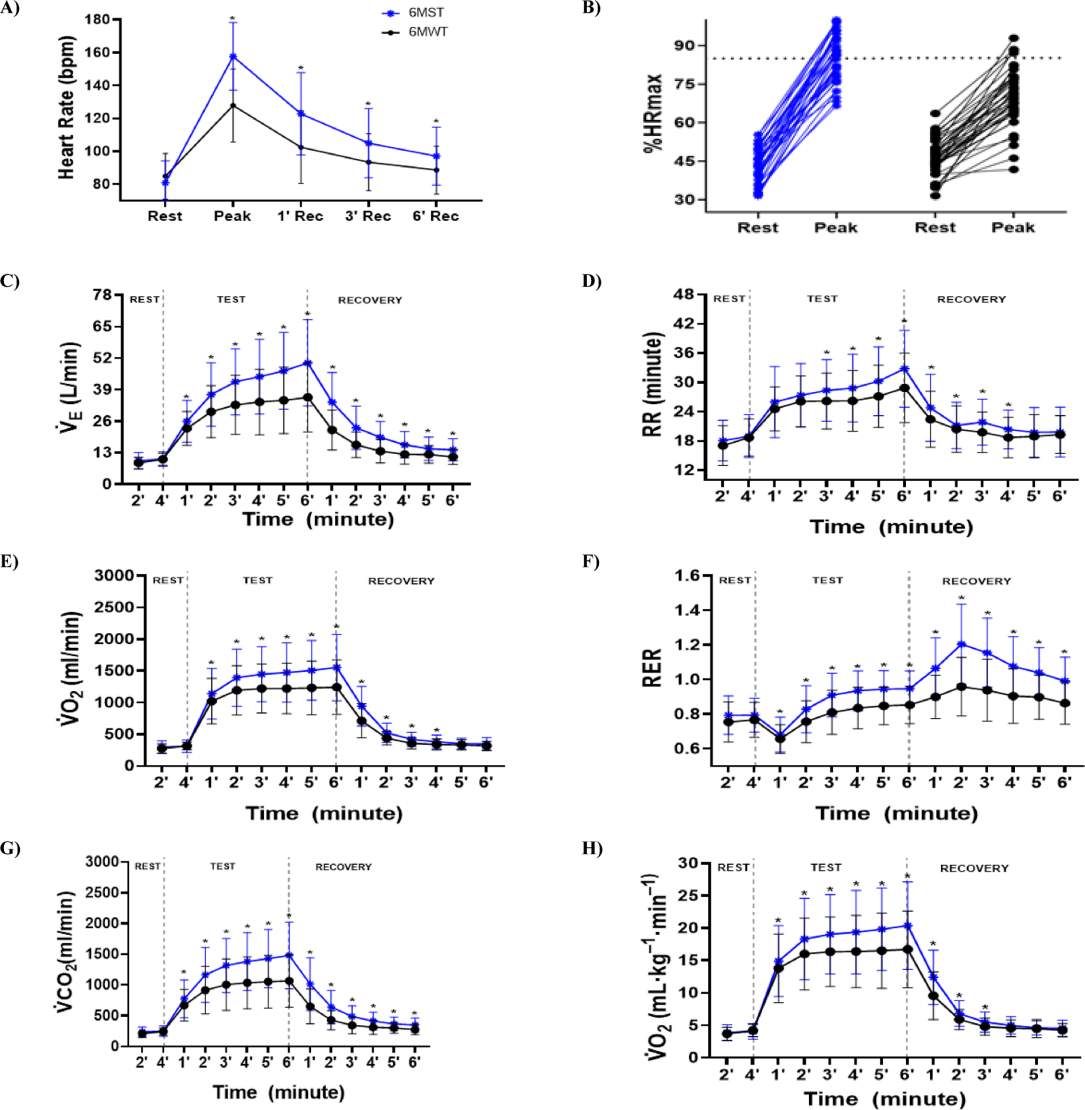
Comparative cardiorespiratory and hemodynamics responses between the 6MST and the 6MWT. 6MST: six-minute step test; 6MWT: six-minute walking test; bpm: beats per minute; rec: recovery; %: percentage; HRmax: maximum heart rate; ${{\dot{\text {V}}}_{\mathrm{E}}}$: minute ventilation; L: liters; min: minute; RR: respiratory rate; ${\dot{\text {V}}}{{\mathrm{O}}_{\mathrm{2}}}$: oxygen uptake; RER: respiratory exchange ratio; ml: milliliter; ${\dot{\text {V}C}}{{\mathrm{O}}_{\mathrm{2}}}$: carbon dioxide production; kg: kilos. *Statistical significance: *p* < 0.05 for Paired t test. The horizontal dashed line in **B** indicates the 85% maximal heart rate threshold.

Significant differences were observed in the hemodynamics responses during the 6MWT and 6MST (Table 2). Peak HR (bpm) was significantly higher during the 6MST (158 ± 21 bpm) compared to the 6MWT (128 ± 22 bpm) (*p* < 0.001; Cohen’s d effect size: 1.395) (Fig. 1A). Recovery HR (bpm) was significantly lower at 1-, 3-, and 6-minutes post-test for the 6MWT (*p* < 0.001) (Fig. 1A). Regarding HRmax (%), the 6MST induced a greater chronotropic response compared to the 6MWT (85 ± 8 vs. 69 ± 11). Individual HRmax (%) data can be seen in Fig. 1B, with a cutoff of 85% in both tests. The 6MST also resulted in higher SBP (*p* < 0.001; Pearson’s r: 0.329), greater respiratory discomfort (*p* < 0.001; Pearson’s r effect size: 0.180) and lower limb fatigue (*p* = 0.015; Pearson’s r effect size: 0.194) as assessed by the BORG scale.

In terms of cardiorespiratory responses, all participants began the tests under similar physiological conditions, with no statistical differences in the baseline measures (Fig. 1C, H). During exercise, responses were significantly more pronounced during the 6MST, with greater ${{\dot{\text {V}}}_{\mathrm{E}}}$ (L/min) (Fig. 1C), ${\dot{\text {V}}}{{\mathrm{O}}_{\mathrm{2}}}$ (mL min) (Fig. 1E), RER (Fig. 1F), ${\dot{\text {V}C}}{{\mathrm{O}}_{\mathrm{2}}}$ (Fig. 1G), and ${\dot{\text {V}}}{{\mathrm{O}}_{\mathrm{2}}}$ (mL kg⁻¹ min⁻¹) (Fig. 1H) from the first minute of exertion and remaining elevated throughout the six-minute test, especially at peak exercise, compared to the 6MWT. The only exception was RR (breaths min⁻¹) (Fig. 1D), which showed a statistically significant difference only from the third minute of the protocol onward. During recovery, variables such as ${{\dot{\text {V}}}_{\mathrm{E}}}$ (L/min) (Fig. 1C), RER (Fig. 1F), and ${\dot{\text {V}C}}{{\mathrm{O}}_{\mathrm{2}}}$ (Fig. 1G) remained significantly higher in the 6MST compared to the 6MWT. On the other hand, the variables RR (breaths min⁻¹) (Fig. 1D), ${\dot{\text {V}}}{{\mathrm{O}}_{\mathrm{2}}}$ (mL min⁻¹) (Fig. 1E), and ${\dot{\text {V}}}{{\mathrm{O}}_{\mathrm{2}}}$ (mL kg⁻¹ min⁻¹) (Fig. 1H) no longer showed a statistically significant difference from the fourth minute of recovery onwards.

### Predictive factors of 6MWT and 6MST performance

Univariate linear regression analyses for variables predicting 6MWT distance, 6MST steps and ${\dot{\text {V}}}{{\mathrm{O}}_{\mathrm{2}}}$ peak (mL kg⁻¹ min⁻¹) during both tests are summarized in the Tables 1 and 2 of the Supplementary Material.

Multiple linear regression analyses for the number of steps during the 6MST and ${\dot{\text {V}}}{{\mathrm{O}}_{\mathrm{2}}}$ peak (mL kg⁻¹ min⁻¹) in both tests are presented in Table 3. For the number of steps during the 6MST, key predictive factors included maximum heart rate, FEV_1_, right lower limb fat percentage, body fat percentage, BMI, and DLcoSB (all *p* < 0.05). For ${\dot{\text {V}}}{{\mathrm{O}}_{\mathrm{2}}}$, the number of steps, sex, age, FEV_1_, and height were significant predictors in models 1–3 (*p* < 0.05). The adjusted R² for the step count models ranged from 0.480 to 0.592, while for ${\dot{\text {V}}}{{\mathrm{O}}_{\mathrm{2}}}$ peak (mL kg⁻¹ min⁻¹), the adjusted R² was between 0.467 and 0.534.

**Table 3:** Multiple linear regression of factors potentially associated with 6MST performance and ${\dot{\text {V}}}{{\mathrm{O}}_{\mathrm{2}}}$ (mL kg^–1^ min^–1^ in mild post-COVID individuals.

Model	Variables	Non-standard coefficients		t	*P* value	Collinearity statistics		Adjusted *R*²	ANOVA *p* value	Durbin-Watson
		β	Error			Tolerance	VIF			
1	Number of steps									
	Constant	− 63.979	74.314	− 0.861	0.395			0.505	< 0.001*	1.962
	Maximum heart rate (bpm)	0.956	0.370	2.584	0.014*	0.900	1.111			
	FEV_1_ (L)	24.867	8.499	2.926	0.006*	0.502	1.994			
	Body fat (%)	− 1.306	0.479	− 2.726	0.010*	0.611	1.636			
	Sex (0, female; 1, male)	− 21.976	11.682	− 1.881	0.068	0.517	1.933			
2	Number of steps									
	Constant	− 76.253	66.103	− 1.154	0.256			0.564	< 0.001*	1.878
	Maximum heart rate (bpm)	1.007	0.337	2.991	0.005*	0.958	1.044			
	FEV_1_ (L)	19.616	6.003	3.268	0.002*	0.886	1.129			
	Right lower limb fat (%)	− 0.191	0.052	− 3.689	0.001*	0.874	1.144			
3	Number of steps									
	Constant	− 85.234	65.396	− 1.303	0.201			0.579	< 0.001*	1.857
	Maximum heart rate (bpm)	1.072	0.330	3.253	0.002*	0.965	1.036			
	FVC (L)	16.050	4.568	3.513	0.001*	0.959	1.043			
	Right lower limb fat (%)	− 0.210	0.049	− 4.255	< 0.001*	0.930	1.075			
4	Number of steps							0.592	< 0.001*	1.835
	Constant	− 80.459	63.814	− 1.261	0.216					
	Maximum heart rate (bpm)	1.000	0.331	3.022	0.005*	0.928	1.078			
	Right lower limb fat (%)	− 0.230	0.048	− 4.816	< 0.001*	0.960	1.042			
	DLcoSB (%)	0.862	0.255	3.376	0.002*	0.906	1.104			
	Sex (0, female; 1, male)	7.190	7.980	0.901	0.374	0.913	1.095			
5	Number of steps									
	Constant	− 119.348	80.515	− 1.482	0.147			0.480	< 0.001*	1.844
	Maximum heart rate (bpm)	1.271	0.386	3.294	0.002*	0.870	1.149			
	BMI (kg/m²)	− 1.886	0.845	− 2.233	0.032*	0.815	1.227			
	FEV1 (L)	11.217	9.251	1.213	0.233	0.445	2.246			
	Handgrip strength (kgf)	1.534	0.846	1.813	0.078	0.482	2.073			
1	${\dot{\text {V}}}{{\mathrm{O}}_{\mathrm{2}}}$ (mL kg^–1^ min^–1^)									
	Constant	− 16.161	17.191	− 0.940	0.353			0.476	< 0.001*	1.846
	Number of steps	0.049	0.021	2.309	0.027*	0.903	1.108			
	Sex (0, female; 1, male)	4.736	2.186	2.167	0.037*	0.465	2.151			
	Height (cm)	0.167	0.109	1.537	0.133	0.435	2.299			
2	${\dot{\text {V}}}{{\mathrm{O}}_{\mathrm{2}}}$ (mL kg^–1^ min^–1^)									
	Constant	− 10.494	17.773	− 0.590	0.559			0.467	< 0.001*	1.779
	Sex (0, female; 1, male)	4.718	2.272	2.077	0.045*	0.438	2.285			
	Height (cm)	0.219	0.106	2.062	0.047*	0.460	2.172			
	Age (years)	− 0.156	0.066	− 2.374	0.023*	0.917	1.091			
	Rest heart rate (bpm)	− 0.025	0.039	− 0.652	0.519	0.869	1.150			
3	${\dot{\text {V}}}{{\mathrm{O}}_{\mathrm{2}}}$ (mL kg^–1^ min^–1^)									
	Constant	12.387	5.096	2.431	0.020*			0.534	< 0.001*	1.993
	Sex (0, female; 1, male)	4.776	1.870	2.553	0.015*	0.565	1.771			
	Age (years)	− 0.092	0.062	− 1.484	0.147	0.889	1.125			
	FEV_1_ (L)	3.695	1.345	2.748	0.009*	0.561	1.784			
	mMRC (0, 1, 2, 3, 4)	− 1.084	0.691	− 1.569	0.126	0.966	1.035			

β: beta; VIF: variance inflation factor; bpm: beats per minute; FEV_1_: forced expiratory volume in first second; %: percentage; DLcoSB: diffusing capacity of the lung for carbon monoxide single-breath; BMI: body mass index; kg: kilos; m: meter; kgf: kilos-force. *Statistical significance (*p* < 0.05).

With respect to predictive analyses, the distance walked during the 6MWT, height and handgrip strength were significant predictors (Table 4), but these variables explained only a small percentage of the variance in performance, with adjusted R² values ranging from 0.125 to 0.153. For predicting ${\dot{\text {V}}}{{\mathrm{O}}_{\mathrm{2}}}$ peak (mL kg⁻¹ min⁻¹), the distance walked and sex were significant, but these models accounted for only a modest portion of the variance in ${\dot{\text {V}}}{{\mathrm{O}}_{\mathrm{2}}}$ peak (mL kg⁻¹ min⁻¹) (adjusted R² between 0.359 and 0.400).

**Table 4:** Multiple linear regression of factors potentially associated with 6MWT performance and ${\dot{\text {V}}}{{\mathrm{O}}_{\mathrm{2}}}$ (mL kg^–1^ min^–1^ in mild post-COVID individuals.

Model	Variables	Non-standard coefficients		t	P value	Collinearity statistics		Adjusted R²	ANOVA p value	Durbin-Watson
		β	Error			Tolerance	VIF			
1	Distance walked (m)									
	Constant	− 250.189	248.668	− 1.006	0.321			0.153	0.018*	2.165
	Height (cm)	3.323	1.369	2.427	0.020*	1.000	1.000			
	Kco [ml/(min mmHg)]	95.018	54.894	1.731	0.092	1.000	1.000			
2	Distance walked (m)									
	Constant	401.029	67.302	5.959	< 0.001*			0.125	0.032*	2.123
	Handgrip strength (kgf)	4.928	1.998	2.466	0.018*	0.978	1.023			
	Age (years)	− 1.924	1.228	− 1.566	0.126	0.978	1.023			
1	${\dot{\text {V}}}{{\mathrm{O}}_{\mathrm{2}}}$ (mL kg^–1^ min^–1^)									
	Constant	− 1.075	4.981	− 0.216	0.830			0.365	< 0.001*	2.423
	Distance walked (m)	0.029	0.007	4.069	< 0.001*	0.945	1.058			
	Sex (0, female; 1, male)	2.866	1.373	2.088	0.044*	0.947	1.056			
	BMI (kg/m²)	0.123	0.120	1.025	0.312	0.976	1.024			
2	${\dot{\text {V}}}{{\mathrm{O}}_{\mathrm{2}}}$ (mL kg^–1^ min^–1^)									
	Constant	4.069	3.733	1.090	0.283			0.359	< 0.001*	2.196
	Distance walked (m)	0.029	0.007	4.051	< 0.001*	0.910	1.099			
	Sex (0, female; 1, male)	3.464	1.629	2.126	0.040*	0.679	1.473			
	DLcoSB [ml/(min mmHg)]	− 0.090	0.109	− 0.830	0.412	0.649	1.540			
3	${\dot{\text {V}}}{{\mathrm{O}}_{\mathrm{2}}}$ (mL kg^–1^ min^–1^)									
	Constant	− 10.624	7.269	− 1.462	0.153			0.400	< 0.001*	2.404
	Distance walked (m)	0.028	0.007	4.095	< 0.001*	0.943	1.061			
	Sex (0, female; 1, male)	3.393	1.368	2.481	0.018*	0.902	1.109			
	FEV_1_ (%)	0.093	0.053	1.761	0.087	0.937	1.067			
	BMI (kg/m²)	0.153	0.118	1.302	0.201	0.955	1.047			

β: beta; VIF: variance inflation factor; bpm: beats per minute; FEV_1_: forced expiratory volume in first second; %: percentage; DLcoSB: diffusing capacity of the lung for carbon monoxide single-breath; BMI: body mass index; kg: kilos; m: meter; kgf: kilos-force. *Statistical significance (*p* < 0.05).

## Discussion

### Main findings

This study aimed to investigate functional capacity, cardiorespiratory and hemodynamic responses, as well as predictive factors associated with the 6MST and 6MWT in individuals recovering from mild COVID-19. Our main findings were: (1) participants achieved, on average, more than 80% of predicted values on both tests; (2) the 6MST elicited greater hemodynamic and cardiorespiratory responses, as well as higher perceived respiratory discomfort and lower limb fatigue, compared to the 6MWT; and (3) multiple linear regression models incorporating anamnesis and clinical assessment variables successfully predicted functional performance and ${\dot{\text {V}}}{{\mathrm{O}}_{\mathrm{2}}}$ peak (mL kg⁻¹ min⁻¹) on both tests.

### Functional performance and physiological responses during the 6MST and 6MWT

Although both the 6MST and 6MWT are submaximal, safe, and well-tolerated tests, the 6MST elicits greater hemodynamic and cardiorespiratory responses. Notably, ${\dot{\text {V}}}{{\mathrm{O}}_{\mathrm{2}}}$ peak (mL kg⁻¹ min⁻¹) during the 6MST was only marginally predicted by the ${\dot{\text {V}}}{{\mathrm{O}}_{\mathrm{2}}}$ peak (mL kg⁻¹ min⁻¹) during the 6MWT (explaining less than 10% of the variance) and only 16% of the variance of 6MST performance was explained by 6MWT distance, further reinforcing the physiological distinction between the two exercise protocols. Although a 30-minute rest interval was provided between the tests, residual fatigue from the 6MWT may have influenced 6MST performance. Additional potential sources of biases related to test order include transient muscular discomfort, carry-over cardiovascular effects, or variations in participant pacing or motivation. Because each test was performed only once, potential learning or familiarization effects could not be evaluated.

Prior studies comparing variations of steps tests to the 6MWT support our findings^53,55,56^. For example, the three-minute step test has demonstrated greater cardiorespiratory load and leg fatigue in both healthy individuals and patients with chronic obstructive pulmonary disease^53,54^. Among patients with coronary artery disease, it was deemed inappropriate to replace the 6MWT^55^ with a two-minute step test, while in individuals with systolic heart failure, the step test was well tolerated and may serve as an alternative^56^. Even in healthy and sedentary populations, the 6MST and 6MWT elicit different physiological demands, with 6MST requiring greater energy expenditure^57^.

It is also important to note that the cohort was relatively young (35 ± 12 years) and largely free of chronic conditions. Physiological responses to exercise can differ markedly with aging and the presence of comorbidities, which may affect both hemodynamic regulation and recovery patterns. In older or clinically compromised individuals, these mechanisms are typically less efficient, potentially resulting in greater cardiovascular stress, slower recovery, and reduced functional reserve. Moreover, the 6MST imposes a higher cardiovascular and metabolic load, whereas the 6MWT more closely reflects daily walking activities and provides a direct measure of functional mobility. Therefore, when applying for these tests in frail or high-risk populations, a careful assessment of the risks and benefits is warranted to ensure safety and the appropriateness of the selected protocol.

### Predictors of 6MST and 6MWT performance and oxygen uptake

Several predictive equations have been developed to estimate functional capacity based on submaximal test performance in diverse populations. These models aim to reduce logistical and financial burdens, especially when standard field or laboratory assessments are not feasible^15,18,20,45,58^.

In our regression models, conventional predictors (age, sex, BMI, height, maximum heart rate and FEV_1_), were significantly associated with 6MST and 6MWT performance, as well as ${\dot{\text {V}}}{{\mathrm{O}}_{\mathrm{2}}}$. Notably, novel predictors identified in our study (DLcoSB, total body fat, and lower limb fat percentage) were also significantly associated with these outcomes. These findings suggest that incorporating variables related to pulmonary diffusion and body composition may enhance the predictive accuracy of models estimating functional capacity and ${\dot{\text {V}}}{{\mathrm{O}}_{\mathrm{2}}}$ peak (mL kg⁻¹ min⁻¹). However, some multiple linear regression models demonstrated relatively low adjusted R² values (< 20%), particularly those predicting the 6MWT distance and corresponding ${\dot{\text {V}}}{{\mathrm{O}}_{\mathrm{2}}}$ peak, indicating limited clinical applicability and warranting cautious interpretation. Although FEV₁ and DLcoSB both reflect pulmonary function, they capture different physiological mechanisms (airflow limitation versus gas exchange efficiency, respectively). In some individuals, FEV₁ may remain within normal limits, indicating preserved airflow, while DLcoSB is reduced, revealing an underlying impairment in oxygen transfer that may constrain exercise performance. This dissociation may be particularly relevant among individuals with prior COVID-19, who may exhibit normal spirometric results despite persistent reductions in pulmonary diffusion capacity. Body composition, assessed through bioelectrical impedance, offerts additional insights by quantifying muscle mass, lean mass, and fat mass, factors that influence oxygen delivery, muscular workload and metabolic efficiency. These variables contribute to explaining interindividual differences in functional capacity and underscore the multifactorial nature of exercise performance, integrating pulmonary, muscular, and metabolic determinants.

For the 6MWT, prior reference equations have explained 15.9% to 78% of the variance in walked distance and up to 75% of peak VO₂ during cardiopulmonary exercise testing (CPET) in the adults^59–61^. These equations commonly involve sociodemographic, anthropometric (weight, height, BMI), pulmonary function (FEV1), muscle strength (peripheral and respiratory), and hemodynamic variables (e.g., HR and SBP)^31,62–66^.

In healthy populations, predictive models for 6MST performance have explained approximately 42–50% of the total variance using variables such as age, sex, abdominal circumference, height and weight^19^. Similarly, ${\dot{\text {V}}}{{\mathrm{O}}_{\mathrm{2}}}$ peak (mL kg⁻¹ min⁻¹) during a modified incremental step test was explained by up to 80% using sex, age, weight and the number of steps^67^. Among individuals with chronic heart failure, the number of steps during the 6MST explained 51% of peak ${\dot{\text {V}}}{{\mathrm{O}}_{\mathrm{2}}}$ during CPET^15^, while in individuals with obesity, BMI, age, and step count explained up to 81% of peak ${\dot{\text {V}}}{{\mathrm{O}}_{\mathrm{2}}}$ variance^58^.

## Clinical impact

In populations with the potential for cardiopulmonary dysfunction, such as COVID-19, accurate estimation of functional capacity and understanding exercise-induced physiological responses are critical. However, clinicians should appreciate the 6MST and 6MWT evaluating different aspects of functional tolerance and physiological stress. Our study contributes novel insights by: (1) providing the first comparative analysis of submaximal test responses in individuals post-mild COVID-19; (2) identifying additional predictors, such as DLcoSB and body fat distribution, that improve prediction of functional performance and ${\dot{\text {V}}}{{\mathrm{O}}_{\mathrm{2}}}$; (3) from a clinical perspective, both the 6MWT and the 6MST provide valuable and complementary information for assessing functional capacity and guiding post-COVID rehabilitation. The 6MWT has been more extensively studied over the years, with well-established cut-off points, prognostic value, and strong evidence supporting its use across a wide range of clinical populations^2^. In contrast, the 6MST offers practical advantages such as lower space requirements, ease of administration, and suitability for remote assessments, particularly in the current era of telerehabilitation, where accessible, safe, and easily supervised tests are increasingly relevant^68^. Ultimately, the choice between the two should be guided by the patient’s clinical condition, rehabilitation goals, and available resources.

## Limitations

Despite rigorous methodology, our study has limitations. The relatively small sample size and absence of a control group limit the generalizability of the findings and preclude direct attribution of observed physiological differences to the effects of COVID-19. A post-hoc power analysis using VO₂ peak indicated that the sample was sufficient to detect moderate effects; however, given the exploratory nature of the study, it may be underpowered to detect smaller effects. No sample size calculation was performed for other cardiorespiratory outcomes, which were analyzed as exploratory measures. Furthermore, external validation of our predictive equations is necessary before they can be applied more broadly. Caution is warranted in extrapolating these findings to populations with different sociodemographic characteristics or varying severities of illness due to COVID-19. Another limitation of this study is the use of reference equations specifically developed for the Brazilian population (Britto et al.^45^ for the 6MWT and Albuquerque et al.^18^ for the 6MST). The choice of these equations was based on their methodological adequacy, cultural relevance, and predictive performance. Although these equations are appropriate for the studied population, they may limit the generalizability of our findings to populations with different characteristics.

## Conclusion

Although the 6MST and 6MWT yielded comparable results in terms of predicted performance percentages, they represent distinct physiological demands. The 6MST elicits greater hemodynamic and cardiorespiratory stress, reflecting higher metabolic and ventilatory requirements. Incorporating novel predictors related to pulmonary diffusion and body composition enhanced the explanatory power of regression models for functional performance and ${\dot{\text {V}}}{{\mathrm{O}}_{\mathrm{2}}}$ peak (mL kg⁻¹ min⁻¹). The choice between these tests should be guided by the specific assessment goals and the individual clinical condition, emphasizingtheir potential complementary use. Furthermore, these findings highlight the the need for external validation of the prediction equations.

## Supplementary Information

Below is the link to the electronic supplementary material.

Supplementary Material 1

## Data Availability

The datasets used and/or analyzed during the current study are available from the corresponding author on reasonable request.
