# Supplementary material for: Comparing hemodynamic and cardiorespiratory responses during six-minute walk and step tests in mild acute COVID-19
Source: Sci Rep. 2026 Feb 23;16:10234. doi: 10.1038/s41598-026-41337-1 (PMC13031849; doi:10.1038/s41598-026-41337-1)
Supplement: Supplementary file 1 — Supplementary Material 1 [file 41598_2026_41337_MOESM1_ESM.docx]

**Supplementary Material**

**COMPARING HEMODYNAMIC AND CARDIORESPIRATORY RESPONSES DURING SIX-MINUTE WALK AND STEP TESTS IN MILD ACUTE COVID-19**

Aldair Darlan Santos-de-Araújo^1,2^, Daniela Bassi-Dibai^3,4^, Renan Shida Marinho^1,5^, Shane A. Phillips^6,7^, Ross Arena^6,7,8^, Audrey Borghi-Silva^1,5,*^

^1^Physical Therapy Department, Cardiopulmonary Physical Therapy Laboratory, Federal University of São Carlos, São Carlos, SP – Brazil.

^2^Paranaense University (UNIPAR), Francisco Beltrão Campus – PR, Brazil.

^3^Department of Dentistry, CEUMA University, São Luís, MA – Brazil.

^4^Postgraduate Program in Management in Health Programs and Services, CEUMA University, São Luís – MA, Brazil.

^5^Postgraduate Program Inter-units of Bioengineering, University of São Paulo, São Carlos – SP, Brazil.

^6^Department of Physical Therapy, College of Applied Health Sciences, University of Illinois at Chicago, Chicago, IL, USA

^7^Healthy Living for Pandemic Event Protection (HL – PIVOT) Network, Chicago, IL, USA

^8^HealthPartners Institute, Minneapolis, MN, USA.

***Corresponding author:**

Audrey Borghi-Silva. Federal University of São Carlos, Cardiopulmonary Physiotherapy Laboratory, Physical Therapy Department, Rodovia Washington Luiz, Postal Code - 13565-905, São Carlos – SP, Brazil. E-mail: [audrey@ufscar.br](mailto:audrey@ufscar.br).

**METHODOLOGY**

**Calibration and Measurement of Oxygen Uptake and Ventilatory Parameters**

Prior to each test, the carbon dioxide (CO2) and oxygen (O2) analyzers underwent calibration with a calibration gas mixture [5% CO2, 12% O2, and nitrogen (N2) balance] along with a reference gas, which was room air corrected to standard temperature and pressure, dry (STPD). The pneumotachograph was calibrated using a known flow generator, ensuring accurate measurement of respiratory parameters during the tests.

**RESULTS**

**Table 1.** Univariate regression analysis of factors potentially associated with 6MST performance and V̇O_2_ (mL·kg^–1^·min^–1^) in mild post-COVID individuals.

| **VARIABLES** | **Number of steps** | | | | **V̇O_2_ (mL·kg^–1^·min^–1^)** | | | |
| --- | --- | --- | --- | --- | --- | --- | --- | --- |
|  | **Non-standardized coefficients** | | **Adjusted R^2^** | **P value** | **Non-standardized coefficients** | | **Adjusted R^2^** | **P value** |
|  | **β** | **Erro** |  |  | **β** | **Erro** |  |  |
| Age (years) | -1.419 | 0.452 | 0.185 | 0.003*† | -0.118 | 0.085 | 0.023 | 0.174† |
| Sex (0, female; 1: male) | 12.946 | 11.916 | 0.005 | 0.284 | 7.900 | 1.645 | 0.361 | <0.001*† |
| Height (cm) | 1.089 | 0.554 | 0.069 | 0.056† | 0.386 | 0.078 | 0.374 | <0.001*† |
| Body mass (kg) | -0.563 | 0.336 | 0.044 | 0.103† | 0.025 | 0.060 | -0.022 | 0.680 |
| Body fat mass (kg) | -1.566 | 0.410 | 0.258 | <0.001*† | -0.201 | 0.077 | 0.131 | 0.012*† |
| Skeletal muscle mass (kg) | 1.168 | 0.854 | 0.022 | 0.180† | 0.467 | 0.130 | 0.232 | 0.001*† |
| Basal metabolic rate (Kcal) | 0.027 | 0.024 | 0.006 | 0.275 | 0.014 | 0.004 | 0.261 | 0.007*† |
| Body fat (%) | -1.794 | 0.454 | 0.273 | <0.001*† | -0.307 | 0.078 | 0.268 | <0.001*† |
| Right lower limb fat (%) | -0.273 | 0.060 | 0.339 | <0.001*† | -0.020 | 0.012 | 0.040 | 0.112† |
| Left lower limb fat (%) | -0.279 | 0.062 | 0.327 | <0.001*† | -0.019 | 0.013 | 0.028 | 0.153† |
| BMI (kg/m²) | -2.815 | 0.969 | 0.160 | 0.006*† | -0.351 | 0.176 | 0.071 | 0.053† |
| FVC (L) | 21.000 | 6.096 | 0.218 | 0.001*† | 5.261 | 0.848 | 0.490 | <0.001*† |
| FVC (%) | 0.488 | 0.493 | -0.001 | 0.329 | 0.001 | 0.086 | -0.026 | 0.988 |
| FEV_1_ (L) | 29.402 | 7.238 | 0.284 | 0.001*† | 6.204 | 1.104 | 0.440 | <0.001*† |
| FEV_1_ (%) | 0.574 | 0.465 | 0.013 | 0.225 | 0.007 | 0.082 | -0.026 | 0.929 |
| FEV_1_/FVC | 0.642 | 0.912 | -0.013 | 0.485 | -0.156 | 0.156 | 0.000 | 0.324 |
| DLOCSB [ml/(min*mmHg)] | 2.652 | 0.660 | 0.280 | <0.001*† | 0.543 | 0.103 | 0.406 | <0.001*† |
| DLOCSB (%, predicted) | 1.129 | 0.339 | 0.205 | 0.002*† | 0.181 | 0.060 | 0.174 | 0.004*† |
| Kco [ml/(min*mmHg)] | 12.679 | 7.530 | 0.045 | 0.100† | 1.447 | 1.324 | 0.005 | 0.282 |
| Kco (%) | 0.489 | 0.383 | 0.016 | 0.210 | 0.136 | 0.064 | 0.083 | 0.007*† |
| TLC (%) | 0.302 | 0.359 | -0.008 | 0.405 | -0.009 | 0.062 | -0.026 | 0.880 |
| MIP (%) | 0.255 | 0.202 | 0.015 | 0.214 | 0.018 | 0.035 | -0.019 | 0.616 |
| MEP (%) | -0.005 | 0.287 | -0.026 | 0.986 | -0.033 | 0.049 | -0.014 | 0.50 |
| Level of physical activity | 1.576 | 4.160 | -0.022 | 0.707 | 0.342 | 0.716 | -0.020 | 0.635 |
| mMRC | -6.809 | 5.739 | 0.010 | 0.243 | -1.601 | 0.973 | 0.042 | 0.108† |
| Handgrip strength (kgf) | 1.869 | 0.767 | 0.112 | 0.020*† | 0.433 | 0.124 | 0.224 | 0.001*† |
| Maximum heart rate (bpm) | 1.412 | 0.451 | 0.184 | 0.003*† | 0.118 | 0.085 | 0.023 | 0.174† |
| Number of steps | - | - | - | - | 0.074 | 0.025 | 0.162 | 0.006*† |
| 6MWT (m) | 0.166 | 0.056 | 0.165 | 0.005*† | 0.014 | 0.011 | 0.021 | 0.184 |
| 6MWT (V̇O_2_ (mL·kg^–1^·min^–1^) | 1.890 | 1.114 | 0.046 | 0.098† | 0.417 | 0.187 | 0.092 | 0.032*† |

6MST: six-minute step test; V̇O_2_: oxygen uptake. mL: milliliter; min: minute; β: beta; kg: kilos; cm: centimeter; Kcal: kilocalories; BMI: body mass index; FVC: forced vital capacity; L: liter; %: percentage; FEV_1_: forced expiratory volume in first second; FEV_1_/FVC: ratio between forced vital capacity and forced expiratory volume in first second; DLcoSB: Diffusing capacity of the lung for carbon monoxide single-breath; mmHg: millimeters of mercury; Kco: transfer coefficient for carbon monoxide; TLC: thoracic lung capacity; MPI: maximum inspiratory pressure; MEP: maximum expiratory pressure; mMRC: modified medical research council; kgf: kilogram-force; bpm: beats per minute. *Statistical significance for univariate regression analysis (p<0.05); †Statistical significance for multiple regression analysis (p<0.20).

**Table 2.** Univariate regression analysis of factors potentially associated with 6MWT performance and V̇O_2_ (mL·kg^–1^·min^–1^) in mild post-COVID individuals.

| **VARIABLES** | **Distance walked (m)** | | | | **V̇O_2_ (mL·kg^–1^·min^–1^)** | | | |
| --- | --- | --- | --- | --- | --- | --- | --- | --- |
|  | **Non-standardized coefficients** | | **Adjusted R^2^** | **P value** | **Non-standardized coefficients** | | **Adjusted R^2^** | **P value** |
|  | **β** | **Erro** |  |  | **β** | **Erro** |  |  |
| Age (years) | -1.470 | 1.293 | 0.007 | 0.263 | -0.037 | 0.071 | -0.019 | 0.605 |
| Sex (0, female; 1: male) | 40.997 | 30.654 | 0.020 | 0.189† | 3.892 | 1.577 | 0.115 | 0.018*† |
| Height (cm) | 3.366 | 1.404 | 0.108 | 0.022*† | 0.167 | 0.077 | 0.086 | 0.037*† |
| Body mass (kg) | 0.500 | 0.900 | -0.018 | 0.582 | 0.064 | 0.048 | 0.019 | 0.192† |
| Body fat mass (kg) | -1.049 | 1.240 | 0.007 | 0.403 | -0.018 | 0.068 | -0.024 | 0.790 |
| Skeletal muscle mass (kg) | 4.023 | 2.173 | 0.059 | 0.072† | 0.249 | 0.116 | 0.084 | 0.039*† |
| Basal metabolic rate (Kcal) | 0.112 | 0.061 | 0.058 | 0.074† | 0.008 | 0.003 | 0.111 | 0.020*† |
| Body fat (%) | -1.810 | 1.367 | 0.019 | 0.193† | -0.129 | 0.073 | 0.053 | 0.083† |
| Right lower limb fat (%) | -0.206 | 0.190 | 0.005 | 0.283 | 0.001 | 0.010 | -0.026 | 0.929 |
| Left lower limb fat (%) | -0.217 | 0.197 | 0.005 | 0.278 | 0.005 | 0.011 | -0.020 | 0.628 |
| BMI (kg/m²) | -2.121 | 2.755 | -0.011 | 0.446 | 0.032 | 0.150 | -0.025 | 0.832 |
| FVC (L) | 36.801 | 17.093 | 0.085 | 0.038*† | 2.544 | 0.889 | 0.155 | 0.007*† |
| FVC (%) | -0.354 | 1.293 | -0.024 | 0.786 | .050 | 0.070 | -0.013 | 0.478 |
| FEV_1_ (L) | 51.053 | 20.891 | 0.113 | 0.019*† | 3.001 | 1.115 | 0.138 | 0.011*† |
| FEV_1_ (%) | 0.149 | 1.230 | -0.026 | 0.904 | 0.062 | 0.066 | -0.003 | 0.152† |
| FEV_1_/FVC | 1.232 | 2.370 | -0.019 | 0.606 | -0.049 | 0.129 | -0.022 | 0.705 |
| DLCOSB [ml/(min*mmHg)] | 3.712 | 1.952 | 0.063 | 0.065† | 0.147 | 0.108 | 0.021 | 0.182† |
| DLCOSB (%, predicted) | 1.106 | 0.983 | 0.007 | 0.268 | 0.036 | 0.054 | -0.015 | 0.510 |
| Kco [ml/(min*mmHg)] | 32.925 | 19.518 | 0.045 | 0.100† | 0.410 | 1.094 | -0.023 | 0.710 |
| Kco (%) | 1.798 | 0.971 | 0.059 | 0.072† | 0.046 | 0.054 | -0.008 | 0.408 |
| TLC (%) | -0.207 | 0.939 | -0.025 | 0.826 | 0.058 | 0.050 | 0.009 | 0.252 |
| MIP (%) | 0.433 | 0.530 | -0.009 | 0.419 | 0.021 | 0.029 | -0.013 | 0.480 |
| MEP (%) | 0.031 | 0.744 | -0.026 | 0.967 | -0.029 | 0.040 | -0.013 | 0.480 |
| Level of physical activity | 4.341 | 10.782 | -0.022 | 0.690 | -0.020 | 0.585 | -0.026 | 0.973 |
| mMRC | -11.683 | 15.032 | -0.010 | 0.442 | -0.763 | 0.811 | -0.003 | 0.353 |
| Handgrip strength (kgf) | 4.459 | 2.013 | 0.091 | 0.033*† | 0.238 | 0.109 | 0.087 | 0.036*† |
| Maximum heart rate (bpm) | 1.394 | 1.291 | 0.004 | 0.287 | 0.037 | 0.071 | -0.019 | 0.605 |
| Distance walked (m) | - | - | - | - | 0.031 | 0.007 | 0.314 | <0.001*† |
| 6MST (number of steps) | 1.119 | 0.379 | 0.165 | 0.005*† | 0.037 | 0.022 | 0.046 | 0.098† |
| 6MST (V̇O_2_ (mL·kg^–1^·min^–1^) | 3.224 | 2.384 | 0.021 | 0.184 | 0.277 | 0.124 | 0.092 | 0.032*† |

6MST: six-minute walking test; V̇O_2_: oxygen uptake. mL: milliliter; min: minute; β: beta; kg: kilos; cm: centimeter; Kcal: kilocalories; BMI: body mass index; FVC: forced vital capacity; L: liter; %: percentage; FEV_1_: forced expiratory volume in first second; FEV_1_/FVC: ratio between forced vital capacity and forced expiratory volume in first second; DLcoSB: diffusing capacity of the lung for carbon monoxide single-breath; mmHg: millimeters of mercury; Kco: transfer coefficient for carbon monoxide; TLC: thoracic lung capacity; MPI: maximum inspiratory pressure; MEP: maximum expiratory pressure; mMRC: modified medical research council; kgf: kilogram-force; bpm: beats per minute. *Statistical significance for univariate regression analysis (p<0.05); †Statistical significance for multiple regression analysis (p<0.20).
